# Rhizosphere Microbiome as an Underexplored Resource for Agroecosystem Sustainability: Insights From the Carrot Root Zone

**DOI:** 10.1111/1758-2229.70325

**Published:** 2026-04-01

**Authors:** Alaba Adewole Adebayo, Olubukola Oluranti Babalola

**Affiliations:** ^1^ Food Security and Safety Focus Area, Faculty of Natural and Agricultural Science North‐West University Mmabatho South Africa; ^2^ Department of Life Sciences, Imperial College London, Silwood Park Campus Buckhurst Road, Ascot Berkshire UK

**Keywords:** biodiversity, biotechnology, climate change, *Daucus carota* L., farming practices, horticulture, metagenomics, resilient agroecosystems

## Abstract

Rhizosphere microbiome is critical for nutrient turnover, pathogen suppression, and stress modulation, forming the basis of microbial products relevant to agriculture. However, microbial communities associated with carrot root zone remain relatively underexplored, with limited studies focused beyond descriptive surveys. Here, we synthesise existing information on the structural, functional, and ecological dynamics of the carrot rhizomicrobiome, highlighting its emerging yet underdeveloped mechanistic profiling. Existing literature indicates that carrot‐associated microbes may play a role in nutrient mobilisation, growth promotion, and antagonism. The early proof‐of‐concept works demonstrate that the microbes may gain potential applications in biofertilizers, biostimulants, and biocontrol agents. While these functions are strongly influenced by soil properties, genotype, and management, only a few carrot‐specific isolates/consortia have been multi‐environmentally validated. The limited progress partly reflects the overall underrepresentation of vegetables in microbiome‐based studies, compared to other major crops. We explored the key characteristics, economic, and agricultural significance of the carrot rhizosphere, highlighting its richness with beneficial microorganisms. Among the gaps identified are inadequate functional‐level and field trial, and insufficient multi‐omics integration, which currently limit biotechnological translation. Addressing these gaps through targeted isolation, mechanistic functional and field validation could position carrot rhizosphere microbiome as a valuable yet underexplored resource for enhancing agroecosystem sustainability.

## Graphical abstract

The diagram represents the generalised pathways for the ecological activities mediated by the rhizosphere microbiome within crop‐soil interactions, based on existing literature. The conceptual pathways are adapted for the carrot root‐zone context described in this review. These activities include the transformation of organic matter, promotion of plant growth, modulation of plant stress, and mobilisation of nutrients. The functions, which result from the interactions among soil properties, root exudates, and microbial communities, collectively support plant performance and contribute to agroecosystem functioning.

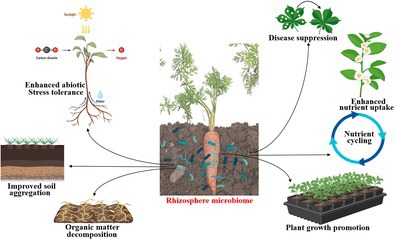

## 1 Introduction

Considering the global developments with regard to increasing urbanisation, growing population with its resulting increase in food demand, and climate change, achieving a sustainable ecosystem remains challenging. To meet the quantity and quality of food required to sustain the increasing global population projected to exceed 9 billion by 2050 (Olanrewaju and Babalola 2022), agriculturists have resorted to different approaches that disrupt the ecosystem's stability (Chukwuneme et al. 2024). The application of fertilisers, pesticides, and herbicides from chemical sources on agricultural soil to stimulate and enhance crop production has been a leading hurdle to attaining a balanced ecological system. As a result, scientists worldwide (Fiodor et al. 2023) have been finding innovative measures to mitigate this menace, utilising eco‐friendly technologies in agricultural research (Rodrigues et al. 2024). The primary goal behind the enormous body of research into sustainable agriculture is to enhance crop productivity, maintain environmental stewardship through reduced or zero reliance on chemical inputs, and improve ecosystem health. Although approaches such as application of engineered disease‐resistant plant species and climate‐change‐resilient plant seeds have proven eco‐friendly and sustainable (Ejaz et al. 2022; Liu et al. 2021; Roy et al. 2023), their adoption is limited by uncertainty of their long‐term effect, reliability, and cost implications (Adedeji et al. 2020). Increasingly, attention is turning towards plant‐associated microorganisms, especially rhizosphere microbiomes, whose ecological functions can be harnessed biotechnologically, in the form of bio‐products (Adedeji and Babalola 2020; Ayangbenro et al. 2022). The narrow region of soil around plant roots, known as the rhizosphere, serves as a hotspot for diverse microbes with unique activities that govern nutrient cycling, soil structure, and plant productivity (Alawiye and Babalola 2019; Enagbonma et al. 2023; Mishra et al. 2021). Research across multiple crops has highlighted the contribution of these microbiomes to diversity and other key ecosystem services essential for sustainability (Aloo et al. 2022; Hussein 2021; Sekhar et al. 2024; Yang et al. 2023). However, the functional capacity of rhizosphere microbiomes in taproot plants, particularly carrot, remains comparatively understudied, creating gaps in designing tailored biotechnological applications for sustainable production.

Carrot (*Daucus carota* L.), a globally cultivated root vegetable with agricultural and economic significance, supports a diverse rhizosphere microbiome, including archaea, bacteria, fungi, and other kingdoms (Adebayo et al. 2025; Singh et al. 2021). Compositions and functioning of these nonhomogeneous microbial communities are influenced by several factors, including root exudates, plant genotype, crop history, agricultural practices, and environmental conditions (Fadiji et al. 2022; Triviño et al. 2023). Although these principles are well established across several crops, including cereals and legumes, the extent to which taproot vegetables, like carrots, follow the same principles remains relatively less resolved. A few studies have reported different context‐dependent abundances of representative beneficial microbes, highlighting how those factors shape microbiome composition and diversity in carrots, below ground (Anderson et al. 2024; Gelaye and Getahun 2024; Triviño et al. 2023). A recent metagenomics study using the Illumina Novaseq X plus platform identified Pseudomonadota, Bacillota, and Actinomycetota as dominant bacterial phyla in carrot rhizosphere under contrasting cropping systems, with soil texture and nutrient content explaining more than 75% of the variance in microbial structure (Adebayo et al. 2025). While there is scarce evidence linking carrot‐associated microbes to plant growth promotion or soil health enhancement, the recent study reported certain functional potentials associated with the carrot rhizosphere. Notably, the metabolic processes, including central carbon metabolism, ATP synthesis, bacteriocin production, resistance mechanisms, dioxygenase activity, and carbon‐nitrogen hydrolase pathways, found to be enriched in the soybean‐precedent system, in the study, are important to agroecological functioning (Chepsergon and Moleleki 2023; Nikitin et al. 2022; Subramanian and Smith 2015). Those context‐dependent functions suggest that carrot rhizosphere communities possess metabolic attributes relevant to plant–soil interaction and nutrient cycling, but their agronomic significance still requires further validation. These findings collectively unveil gaps in the knowledge of system‐specific dynamics that could limit their potential agricultural applications, while positioning the carrot rhizomicrobiome as a currently underexplored resource that aligns with agroecological principles of biodiversity and soil functioning.

This review, therefore, attempts to bridge the gap by discussing current, though limited, evidence on carrot rhizosphere microbiome and evaluating its potential implications for resilient agriculture. We synthesise current knowledge on: (i) composition and ecological drivers of carrot rhizomicrobial communities, (ii) genotype‐management‐microbiome interactions documented for carrot, (iii) their context‐dependent functional potentials, and (iv) the extent to which carrot‐specific rhizosphere microbiome findings can inform biotechnological applications. The review aims to provide an evidence‐based foundation for investigation and potential application of carrot rhizomicrobiome in advancing the achievement of environmental stewardship while pursuing food security. The literature contributes to the emerging knowledge while identifying existing research gaps to harness the potential agroecological role of carrot rhizosphere microbiome for sustainable agroecosystems. It will inform studies and policies in line with the concerted effort of the United Nations Sustainable Development Goals towards zero hunger (SDG 2) and life on land (SDG 15) to benefit humans greatly.

## 2 Ecological Role of Rhizosphere Microbiome in Agroecosystem Functioning

**FIGURE 1:**
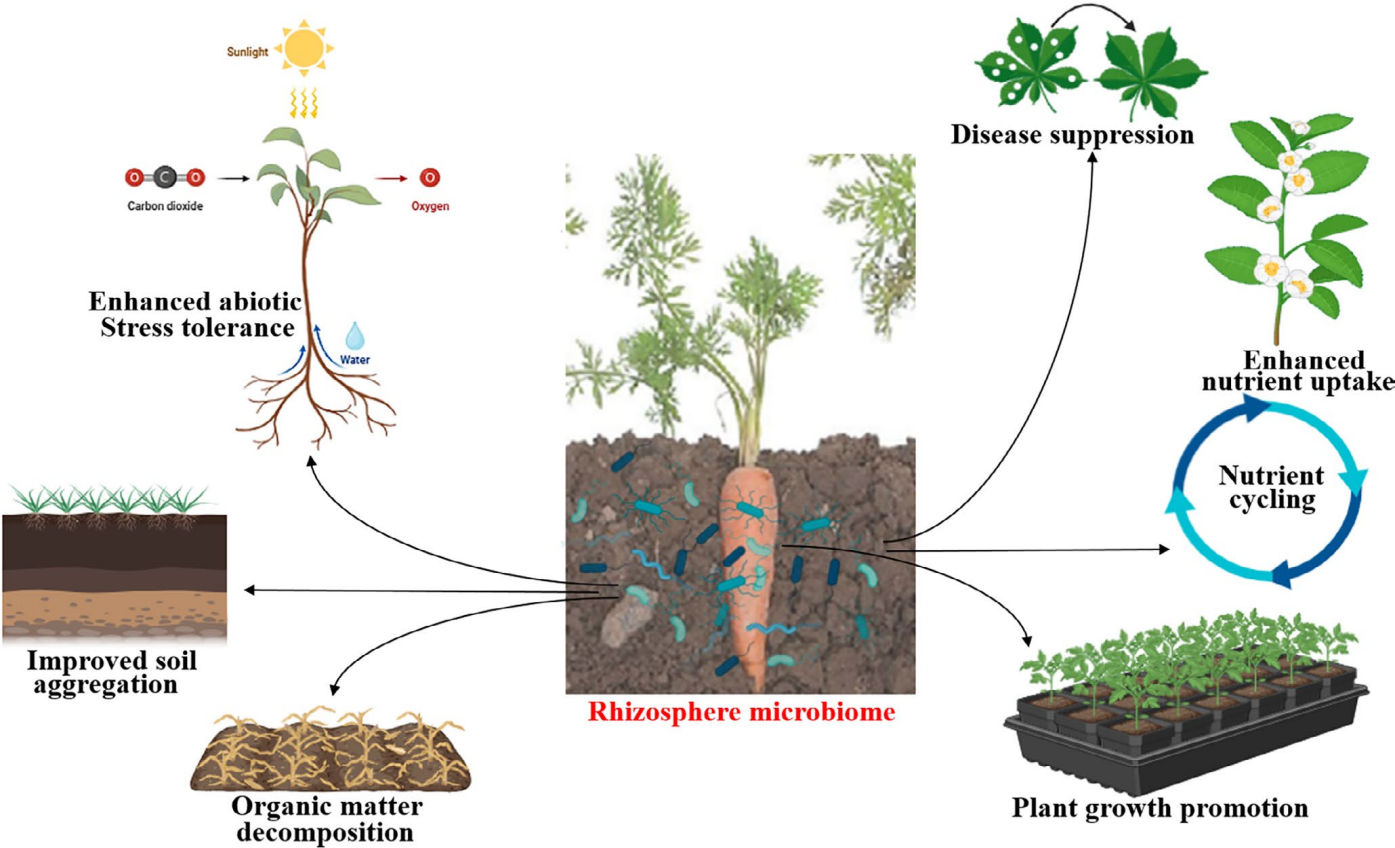
Conceptual representation of rhizosphere microbial contributions to ecological processes within the crop‐soil system. The diagram represents the generalised pathways for the ecological activities mediated by the rhizosphere microbiome within crop‐soil interactions, based on existing literature. The conceptual pathways are adapted for carrot root‐zone context described in this review. These activities include the transformation of organic matter, promotion of plant growth, modulation of plant stress, and mobilisation of nutrients. The functions, which result from interactions among soil properties, root exudates, and microbial communities, collectively support plant performance and contribute to agroecosystem functioning. Created with BioRender.com.

The interconnection between ecosystem processes and agricultural practices, agroecosystems, is based on the fundamental services provided by soil, involving physical, chemical, and biological processes (Notaro et al. 2022). These processes contribute to soil fertility, as well as resource conservation and biodiversity, which interact to promote the quality and productivity of the systems (Dardonville et al. 2022; Estrada‐Carmona et al. 2022). At the core of these interrelationships is microbiome diversity, playing a significant role in soil structural dynamics, nutrient transformation, and root‐soil signalling, which collectively support plant growth and agroecosystem functioning. These microbial‐mediated processes spanning organic matter decomposition, nitrogen fixation, solubilisation, and mobilisation of essential elements are well‐documented across various crops, forming the ecological basis of plant–soil‐microbiome interactions (Dardonville et al. 2022). Notably, because activities of diverse microbes are high at the root, the rhizosphere serves as a biological interface where plant exudates shape microbial recruitment and activity in a way that impacts nutrient availability, stress tolerance, and soil health (Molefe et al. 2023). The role of the rhizosphere communities in a functioning system extends across other key processes, such as disease suppression, carbon turnover, and improved soil aggregation, which fall under regulating and supporting ecosystem services underpinning crop productivity (Villa‐Cox et al. 2023). However, these roles are context‐dependent, as management practices, soil type, cropping history, and plant genotype all shape rhizomicrobiome community and functional architectures, which consequently determine the agronomic outcomes (Oliveira et al. 2024). Knowledge on how these system‐specific factors drive the microbiome structural and functional dynamics, which has attracted significant attention recently, is crucial for achieving robust agricultural productivity with a resilient ecosystem (Estrada‐Carmona et al. 2022). Understanding this interplay across diverse crop varieties and farming practices will contribute immensely to the optimal and efficient biotechnological exploration of microbial communities for sustainable agroecosystems, a long‐time global scientific pursuit. A conceptual summary of the rhizosphere microbiome contribution to ecologically significant processes within the crop‐soil interactions is conceptually illustrated in Figure 1.

**FIGURE 2:**
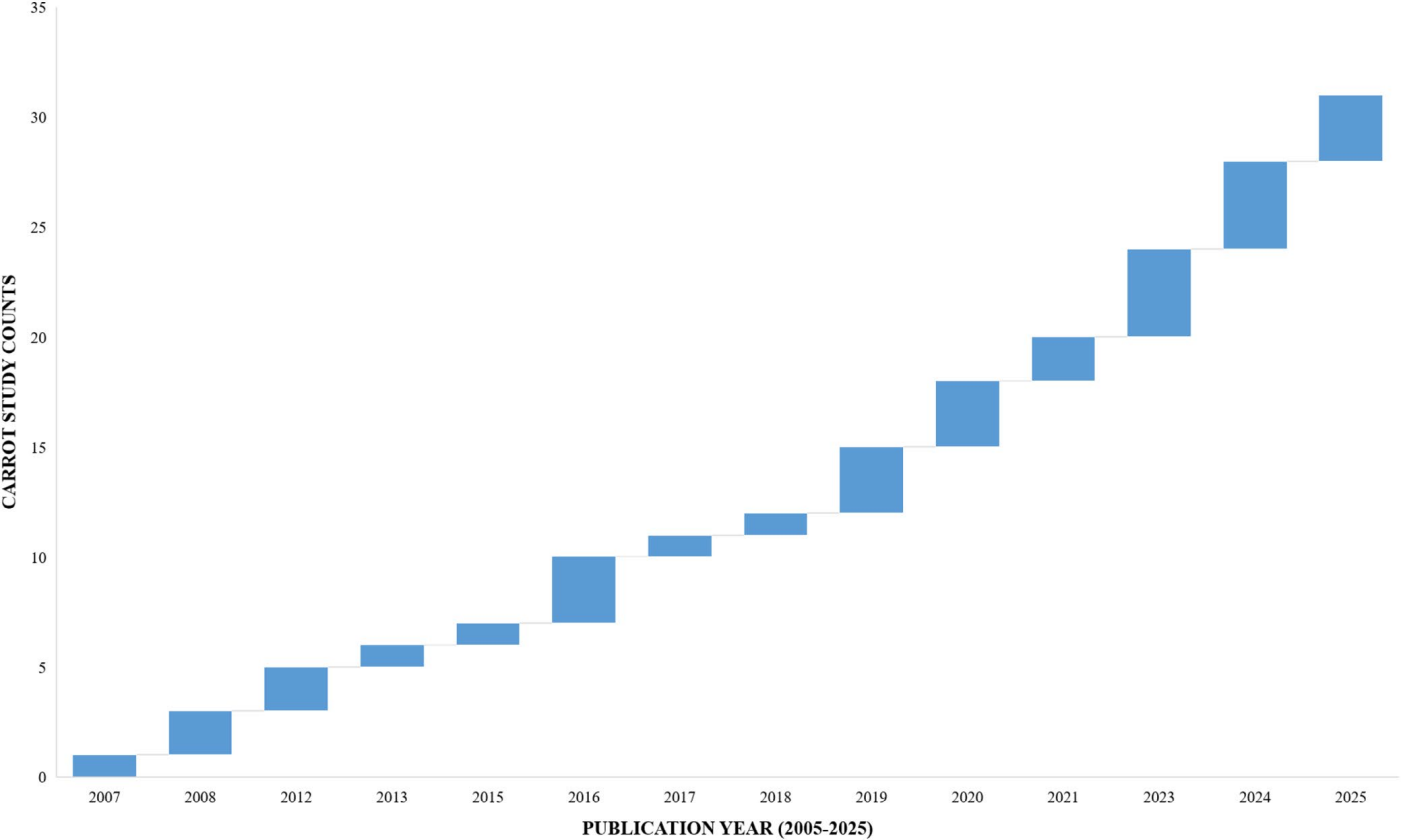
Trend of research attention on carrot rhizosphere microbiome over 2 decades. Counts per year was adopted from the query search result of the WoS Core Collection, spanning 2005 to 2025. Years with missing values do not have a publication record.

Recent developments in sequencing technology and computational biology continue to unravel crop rhizosphere microbiome dynamics under diverse conditions at various scales, underscoring their central role in resilient agroecosystems (Demirel et al. 2024; Li, Zheng, et al. 2024). The dynamics are being studied to understand how diversity, functional redundancy, and adaptive capacity of the microbes contribute to crop production (Aloo et al. 2022). Insights from these studies are currently informing how microbial communities from the rhizosphere are increasingly harnessed for eco‐friendly biotechnological applications, including biostimulants, biofertilizers, biocontrol, and bioremediation agents (Kumar et al. 2022). However, while the microbes from several crops have been translated, studies on such potential in vegetables are comparatively fewer, with more limited fieldwork on their rhizosphere community impact on plant–soil interactions (Philippot et al. 2021). This is particularly evident in carrots, with only a few studies that mostly highlight taxonomic structures and potential functions, and experimental validations remain scarce (Adebayo et al. 2025). A bibliometric comparison using the Web of Science Core Collections (2005–2025) indicates a notable difference in research attention across selected crops, showing thousands of rhizosphere microbiome‐related studies on maize, wheat, and tomatoes (Table 1). In contrast, only a few dozen of these studies focused on carrots, underscoring that the domain, maybe emerging (Figure 2), remains genuinely underexplored. Although the translation of rhizosphere microbiome has recorded marked progress with cereals, legumes, and some vegetables like tomato (Obiazikwor et al. 2025), the emerging carrot‐specific evidence indicates that its rhizosphere microbiomes hold similar opportunities (Ahamad et al. 2023; El‐Tarabily et al. 1997). Apparently, the ecological implications of functionally relevant, though limited, properties of these communities from the carrot rhizosphere are worthy of robust experimental confirmation for potential application (Adebayo et al. 2025; Paparella et al. 2024). Hypothetically, their diversity, plus post‐validation properties, such as plant growth promotion, pest and pathogen suppression, and organic acid degradation essential to plant production (Chepsergon and Moleleki 2023), will make the carrot rhizomicrobiome a promising bio‐resource for sustainable agroecosystem development. Furthermore, there is a need to understand how carrot rhizosphere studies diverge from or are consistent with the broader plant–microbiome principles to better position the crop for modern agricultural benefits.

**TABLE 1:** Comparative visibility of rhizosphere studies across selected crops (2005–2025) (Web of Science; accessed November 23, 2025).

Crop (Scientific name)	Number of publications (2005–2025)	% research outputs	Representative reviews/studies
Major crops			
Maize (Corn) (*Zea mays*)	1335	8.4%	(Hou et al. 2024; Shi et al. 2024)
Wheat (*Triticum aestivum*)	1287	8.1%	(Raheem et al. 2018; Wagi et al. 2023)
Rice (*Oryza sativa*)	958	6.0%	(AL‐Huqail et al. 2025; Luo et al. 2025)
Soybean (*Glycine max*)	545	3.4%	(Agyekum et al. 2025; Qiu et al. 2025)
Vegetable			
Tomato (*Solanum lycopersicum*)	1227	7.7%	(Adedayo et al. 2022; Obiazikwor et al. 2025; Rasheed et al. 2025)
Potato (*Solanum tuberosum*)	320	2.0%	(Mao et al. 2022; Yang et al. 2025)
Cabbage (*Brassica oleracea*)	131	0.8%	(Ping et al. 2024; Zhang et al. 2024)
Carrot (*Daucus carota*)	**31**	**0.2%**	(Adebayo et al. 2025; Zhu et al. 2024)
**Total research outputs**	**15,900**		

*Note:* Counts derived from a topic search in the Web of Science Core Collections, using query search logic: (TS = (“rhizosphere microbiome” OR “rhizosphere microbiota” OR “rhizosphere microbial community” OR rhizomicrobiome OR “rhizosphere‐associated microorganism” OR rhizobacteria OR “rhizosphere microorganism” OR “rhizosphere microbes”)) AND (TI = (rice OR “*oryza sativa*”) ORAB = (rice OR “*oryza sativa*”)). Search, conducted on 23 November 2025, was filtered for only articles and reviews, English language, and timespan 2005–2025. Counts represent indexed records and serve as indicators of relative research attention, reproducible with the recorded query and date. The number and percentage for carrots research were indicated in bold to highlight its comparatively fewer rhizosphere microbiome studies.

## 3 Carrots and Their Rhizospheric Microbiome

### 3.1 General Description and Significance of Carrot Plant

**FIGURE 3:**
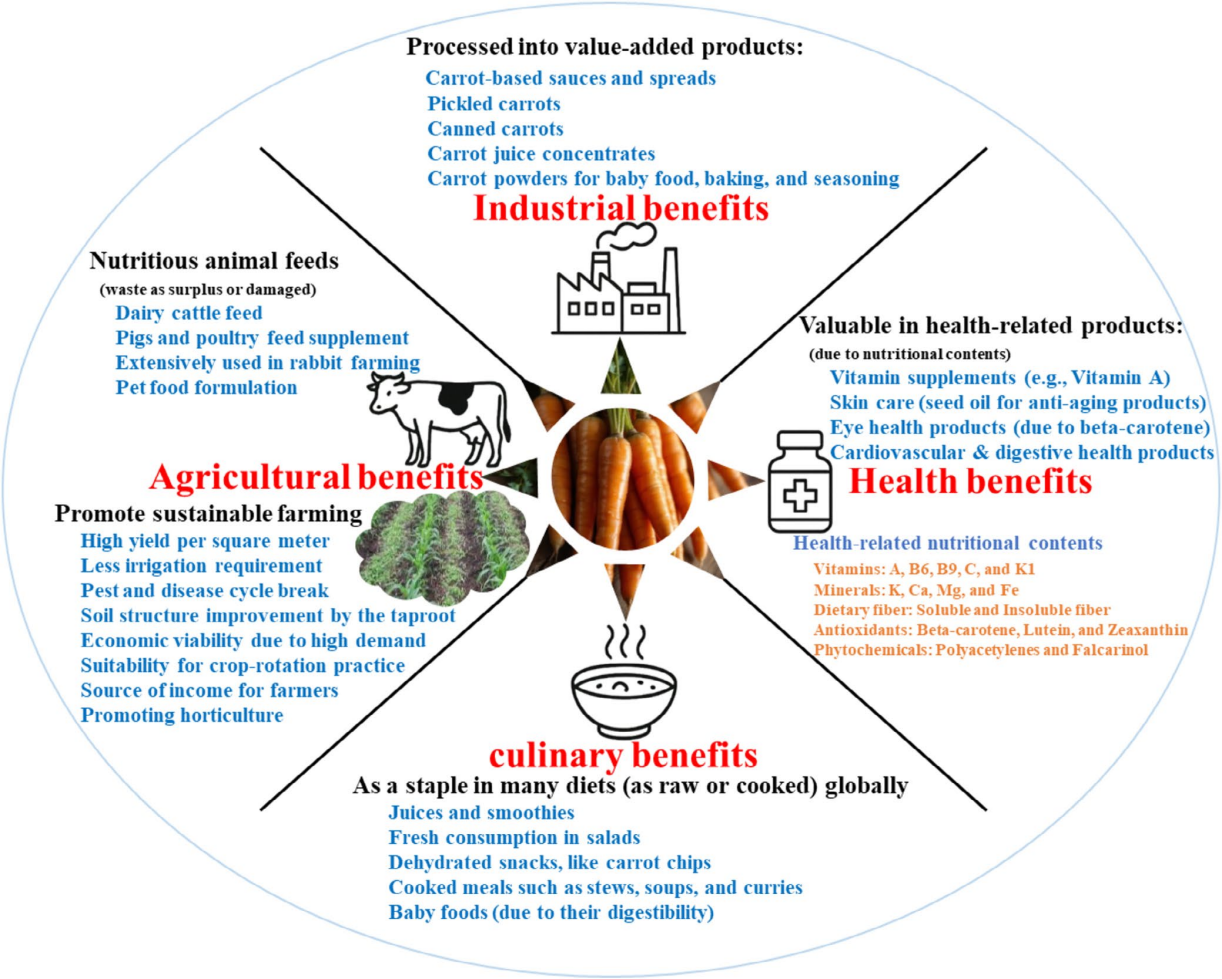
Conceptual overview of the major economic and utilisation pathways of carrots. Diverse application and uses of carrots within multiple sectors, including (i) agricultural uses, such as in livestock feed and farm practice systems; (ii) industrial uses, such as processing into juices, powders, canned products, and condiments; (iii) culinary uses as a widely consumed fresh and processed vegetable; and (iv) health‐related uses, reflecting its nutritional composition and carotenoid content. These categories summarise the broad economic relevance of carrot in horticulture, food industries, and value‐added products. Created with BioRender.com.

Carrot (*Daucus carota* L.) is a biennial root vegetable plant in the Apiaceae family that grows as a taproot. It is widely cultivated for its nutritional value, flavour, and versatility in fresh and processed forms (Godwin et al. 2024; Paparella et al. 2024). This plant is also valued for its micronutrient composition, particularly β‐carotene and other carotenoids that are linked to dietary vitamin A supply (Anjani et al. 2022). Beyond its nutritional role, carrot is an essential commodity that contributes to global agricultural economy, including international trade and horticultural industry (Paparella et al. 2024; Singh et al. 2021). Carrot is one of the most widely cultivated vegetable crops with a growing market value projected to continue at a compound annual growth rate (CAGR) of 4.19% from 2025 to 2035 (https://www.marketresearchfuture.com/reports/carrot‐market‐21806, accessed on 24 November 2025). The global production of carrots and turnips increased from 41.61 Mt. harvested from 11.39 ha in 2021 to 42.04 Mt. harvested from 11.41 ha, with estimate yield increase of 331.9 kg/h in 2022 (FAOSTAT, https://www.fao.org/faostat/en/#data/QCL, accessed on 24 November 2025). The growing records position carrots among major contributors to the vegetable market and food supply chains (Godwin et al. 2024; Singh et al. 2021) worldwide. A quantitative context of agricultural and market relevance, from available global economic indicators for carrot production, is summarised in Table 2. Additionally, as a valuable crop produced year‐round, carrot is cultivated through both intensive and subsistence farming systems, enhancing their horticulture, food, and value‐added benefits (Figure 3) relevant within the global trade dynamics (Singh et al. 2021). It serves as a staple for diversity and specialty in commercialising fresh produce, juices, and processed foods, such as snacks and soup, bolstering its market values (Chaitra et al. 2018; Godwin et al. 2024). Additionally, production and marketing of carrots, particularly in developing countries, involve smallholder farmers who not only benefit from employment opportunities but also rely on the crop for livelihoods (Godwin et al. 2024).

**TABLE 2:** Global production and economic indicators for carrots (FAOSTAT item code 01251: Carrots + turnips).

Indicator	Value (FAO STAT 2023)	
	Carrots	Vegetables
Global production (t)	41,393,537.66	1,186,682,488.52
Global harvested area (ha)	1,127,948	59,131,841
Average global yield (kg/ha)	36,6:8.1	20,068.4
Current gross production value (1000 USD)	16,228,775	645,971,091
Global export volume (t)	3,052,145.2	5,485,736.15*
Global export value (1000 USD)	1,878,123	7,517,609*
Global import volume (t)	2,792,263.07	5,377,224.65*
Global import value (1000 USD)	1,727,097	7,596,426*
Top 5 producers	China, Uzbekistan, United States, Russian Federation, and United Kingdom	China, India, Viet‐Nam, Nigeria, and Philippines

*Note:* The values represent aggregated market estimates for the combined “carrots and turnips” category data (item code 01251) available on the FAOSTAT production and trade domains.

* Values refers to aggregated data for Vegetables (frozen) category.

The economic relevance, cultivation requirements, and biology of carrot, including its interaction with biotic components of the soil, are important considerations for improving crop productivity (Paparella et al. 2024). The biology of carrots is uniquely comprised of morphological traits associated with both vegetative and reproductive phases; it focuses on leaf development and root thickening in the former, while it produces flowering stalks in the latter (Chaitra et al. 2018; Linke et al. 2019; Wohlfeiler et al. 2021). Carrot relies on C3 photosynthetic pathways typical of temperate vegetable crops, enabling the plant to utilise carbon dioxide efficiently (Kyei‐Boahen et al. 2003). The edible taproot length serves as storage for water and essential nutrients, including carbohydrates. It requires cool temperatures (between 15°C and 20°C), adequate water, and nutrients, making it grow best in well‐drained sandy soil rich in organic content (Wohlfeiler et al. 2021). Carrots exist as cultivars distinctly classified into two subspecies: the cultivated species (*Daucus carota* subsp. *sativus*) and wild relatives (*Daucus carota* subsp. *carota*) known as weeds, which serve as genetic resources for crop breeding (Godwin et al. 2024). With 18 diploid chromosomes, carrots exhibit varying size, shape, and colour that categorise them into different types based on these polygenic attributes crucial to disease resistance, nutritional content, and improving flavour in the plant (Chaitra et al. 2018). Carrot cultivation is generally considered less management‐intensive compared to other vegetable crops, especially when planted in loose soil free of stones and debris with adequate sunlight (at least 6 h daily) (Chaitra et al. 2018). Consistent moisture (~25 to 35 mm per week), regular weeding, a balanced nutrient supply based on soil tests, and effective pest and disease control strategies are key to the productive cultivation of carrots (Godwin et al. 2024). Interestingly, due to its short growth period and non‐complex cultivation with moderate input requirements, carrot farming is relatively compatible with different cropping systems, promoting efficient land use (Godwin et al. 2023; Godwin et al. 2024; Paparella et al. 2024). Therefore, continued research into carrot‐associated biotic interactions, including its rhizosphere microbiomes, is essential to clarify their contributions to carrot performance, crop management outcomes, thereby informing modern production practices.

### 3.2 Carrot Rhizosphere: Key Characteristics

The rhizosphere is a complex and dynamic environment surrounding plant roots, characterised by a peak of biological activity and ecological interactions (Adedeji and Babalola 2020). Generally, the components of a rhizosphere work together to modify the physical, chemical, and biological properties of the region, which in turn, play a pivotal role in plant growth, development, and health (Babalola et al. 2021). The structure and composition of this zone are important determinants of balanced conditions, creating air space that enhances nutrient exchange and respiration, as well as influences microbial activity, nutrient availability, and root development (Olanrewaju and Babalola 2022; Wendel et al. 2022). In carrots, several soil‐ and plant‐associated factors influence this zone, including root exudates, temperature, moisture, soil textures, organic matter, and a diverse community of microorganisms (Anderson et al. 2024; Godwin et al. 2024). These factors collectively influence microbial recruitment and activity, driving organic matter transformation and plant root‐soil interactions in carrots (Anderson et al. 2024; Hua et al. 2024).

Carrots are typically cultivated in well‐drained, sandy loam or loamy soil, loose enough to favour deep root penetration and adequate aeration for exudate release (Linke et al. 2019). The organic compounds released into this zone, including organic acids, polysaccharides, amino acids, and phenolic compounds, influence microbial colonisation, nutrient solubilisation, and soil properties (Anderson et al. 2024). Meanwhile, exudates such as polysaccharides enhance soil aggregation and water retention (Brisson et al. 2022), while many others may act as chemoattractants or signal molecules for nutrients and antimicrobial agents, which determine microbiome assembly and their interaction with plants (Hua et al. 2024). The quantity and chemical properties of exudates vary with plant developmental stage, cultivar, and environmental factors, contributing to the heterogeneity reported in carrot rhizosphere studies (Anderson et al. 2024; Bagci et al. 2023; Triviño et al. 2023). All these factors intrinsically interact with the microbial community to form a dynamic carrot rhizosphere environment whose composition has been reported to respond to farm management practices, fertilisation regimes, and cropping systems (Adebayo et al. 2025; Bagci et al. 2023). Several other determining principles that shape the rhizosphere are established across different crops, but carrot‐specific reports are limited and largely descriptive, underscoring the need for mechanistic studies.

#### 3.2.1 Community Structure of Carrot Rhizosphere Microbiome

The rhizosphere microbiome comprises diverse microbial communities that contribute to nutrient transformation and soil processes underpinning plant–soil interactions (Babalola et al. 2021; Fasusi et al. 2023). The communities primarily consist of both soil fauna of varying size classes and microorganisms, including bacteria, archaea, and fungi (Akinola and Babalola 2021). Studies have shown that microorganisms are the major drivers of biochemical processes within rhizosphere zones across crops (Cheng et al. 2023). Some groups of microbes, such as nitrogen‐fixing bacteria and arbuscular mycorrhizal fungi (AMF), mutually benefit plants by enhancing nutrient uptake, as they rely on the plant exudates for sustenance (Fasusi et al. 2023). Many others, including those of the genera *Bacillus* and *Pseudomonas*, are widely recognised in plant‐associated systems for traits linked to growth promotion, nutrient solubilisation, and disease suppression (Chen and Liu 2024). Competition among microbial groups in plant rhizospheres can sometimes lead to the incidence of soil‐borne pathogens in plants, including those associated with carrot diseases like rust and root rot (El‐Tarabily et al. 1997; Yadav et al. 2021).

Across plant species, the dominant rhizosphere microbiome typically includes phyla Proteobacteria, Actinobacteriota, and Firmicutes among bacteria, Ascomycota and Bacidomycota among fungi, and Euryarchaeota and Crenarchaeota among archaea (Agyekum et al. 2023; Fadiji et al. 2021). The carrot rhizosphere has been reported in different studies to contain various microbial communities belonging to these similar groups (Adebayo et al. 2025; Anderson et al. 2024; Bagci et al. 2023). The composition and diversity of this carrot‐associated microbiome vary with changes in biotic and abiotic components driven by different factors such as soil management practices, cropping system, fertilisation regime (Anderson et al. 2024; Bagci et al. 2023). For instance, Rachwal et al. (2021) reported a shift in the bacterial and archaeal phyla assemblage under different agricultural systems, particularly in organic and conventional farming. Similarly, an Amplicon‐based metagenomics study indicates that the carrot rhizosphere commonly harbours bacteria genera such as *Bacillus*, *Geobacillus*, *Candidatus* *Nitrosotalea*, and a number of unclassified taxa within Nitrososphaeraceae, Vicinamibacterales, Gemmatimonadaceae, and Bacillales (Bagci et al. 2023). Although these profiles are consistent with structural patterns common in several crops, there is a need for more mechanistic studies on carrot‐specific rhizosphere microbiome architecture.

### 3.3 Insight Into the Functional Potential of Carrot Rhizosphere Microbiome

Rhizosphere microbial communities contribute to several soil processes essential for nutrient availability and plant–soil interactions (Hartmann and Six 2023). The inter‐ and intra‐communication between the diverse body of microbial communities regulates the biogeochemical activities in the rhizosphere (Fasusi et al. 2023; Hussein 2021). These activities include nutrient cycling, decomposition of organic and inorganic matter, contaminant degradation, pathogen inhibition, and greenhouse gas reduction. Although most mechanistic insights into how these microorganisms direct them are derived from general rhizosphere studies, those processes collectively underpin soil functions relevant to plant performance (Cheng et al. 2023; Notaro et al. 2022).

Generally, the rhizosphere microbiome contributes to the availability and acquisition of nutrients by plants by transforming complex organic matter into accessible forms (Adebayo et al. 2023). Microbial activities such as hyphal and biofilm formation can enhance soil structure and aggregation, as well as influence water retention capability and carbon dynamics in the soil (Ahmad and Li 2021; Tang et al. 2023). Nitrogen‐fixing, potassium‐ and phosphorus‐solubilising microbes can facilitate the bioavailability of essential nutrients for plant growth (Aasfar et al. 2021; Wang, George, et al. 2022). Some of these organisms may contribute to the abundance of micronutrients needed by plants through the degradation of contaminants and heavy metals (Rai et al. 2023). Notably, similar groups of microbes are potentially present in soils where carrots are cultivated, where their activities may affect nutrient availability and plant development. For instance, Zhu et al. (2024) identified *Bacillus firmus* MN3, *Acinetobacter pitti* MP41, and *Bacillus subtilis* PK9 as nutrient‐enhancing bacteria from the rhizosphere of a greenhouse‐grown carrot. Another key player among the microbiome associated with plant rhizosphere is the arbuscular mycorrhizal fungi (AMF), known broadly for enhancing nutrient uptake and modulating plant responses to environmental stresses, such as drought and salinity (Fasusi et al. 2023; Yadav et al. 2021). Similarly, carrot rhizosphere has been reported to be enriched with diverse genera, including *Claroideoglomus*, *Diversispora*, and *Funneliformis*, that belong to the AMF communities, indicating their potential support for carrot growth and performance (Ilyas et al. 2024).

The support that these rhizosphere microbes offer plants may be direct through nutrient mobilisation or via indirect mechanisms involving microbial production of compounds that influence plant defence and stress responses (Adedayo et al. 2022; Adedeji et al. 2020). The direct mechanisms involve releasing stimulating compounds in the form of nutrients that enhance the development of roots and aerial biomass of the plant (Omotayo and Babalola 2021). Some of these nutrients, including phosphorus and potassium, are solubilised, while iron and other micronutrients, such as calcium and magnesium, are chelated, and nitrogen is biologically fixed by the rhizosphere microbiomes (Adedeji and Babalola 2020). Some of the organisms can synthesise hormone compounds, such as gibberellins, auxins, and cytokinins, that stimulate plant cell division (Ayangbenro et al. 2022; Gavelienė et al. 2021). Additionally, microbial‐induced 1‐aminocyclopropane 1‐carboxylate deaminase (ACC) enzyme can reduce ethylene content in the roots, increasing root density and length (Enagbonma et al. 2023). Complementarily, the indirect mechanism involves microbial production of antibiotics, pigments, siderophores, water‐soluble vitamins, volatile compounds, and phytohormone‐like substances (Ayangbenro et al. 2022; Babalola et al. 2021; Nwachukwu and Babalola 2021). These compounds modify the physicochemical and ecological components of the rhizosphere, which may trigger the plant's innate resilience and systemic resistance potential, increasing plant quorum sensing and inhibiting biofilm formation by pathogens (Enagbonma et al. 2023). This process can enhance protection and resilience against biotic and abiotic stressors, contributing to plant yields, quality, and health (Fasusi et al. 2023; Omotayo and Babalola 2021). Enzymes such as chitinases and glucanases, produced by rhizosphere microbiomes, may also degrade fungal cell walls and influence pathogen dynamics (Akanmu et al. 2021).

Several microbial taxa, including *Burkholderia*, *Bacillus*, *Pseudomonas*, and members of Actinobacteriota and mycorrhizal fungi, identified in carrot soils have been reported from other crops to possess beneficial traits (Aloo et al. 2022; Babalola and Akindolire 2011; Petrović et al. 2024). Archaeal groups such as Euryarchaeota and Crenarchaeota have been detected in carrot rhizosphere, but their specific functional role in carrot systems remains underexplored (Adebayo et al. 2025). These microbes are known to contribute to the health and performance of their associated crops through various functions spanning nutrient cycling, growth promotion, and pathogen suppression (El‐Saadony et al. 2022; Linke et al. 2019). However, only a limited number of studies have directly isolated and evaluated carrot‐specific rhizosphere species for growth‐promoting potentials (Zhu et al. 2024). More importantly, there is a report of a sugarcane root‐associated bacterium, *Gluconacetobacter diazotrophicus*, enhancing carrot yield in response to varying nitrogen and phosphorus content (Ceballos‐Aguirre et al. 2023), indicating that functional activities of rhizosphere microbes, although they are factor‐dependent, can be harnessed for enhanced carrot production.

## 4 Drivers of the Rhizosphere Soil Microbiome: The Current State of Carrot Root Zone

The compositional structure and functional potential of carrot rhizosphere microbes are influenced by various interacting factors, including soil properties, climatic conditions, plant genotype, and agricultural management practices (Anderson et al. 2024; Mendes et al. 2013; Triviño et al. 2023; Turner et al. 2013). Through a dynamic interplay, these interconnected drivers determine which microbial communities are recruited and which metabolic functions are employed, as observed in rhizosphere studies across different crops and in the limited but growing carrot literature (Anderson et al. 2024; Bagci et al. 2023; Meena et al. 2023; Mendes et al. 2013). Understanding these dynamics is crucial for designing field experiments and management practices that evaluate rhizosphere microbial functions that potentially benefit carrot production.

### 4.1 Soil Type

The microbial communities inhabiting plant root zones are strongly influenced by the physical and chemical properties of the soil, including organic matter, pH level, nutrient content, and texture (Philippot et al. 2013; Triviño et al. 2023). Variation in these components shapes the rhizosphere environment, allowing for the selection of different microbial taxa with specific functions (Triviño et al. 2023; Turner et al. 2013). Interactions between these soil properties not only drive microbial diversity but also impact the metabolic functions of the communities within plant root zones (Anderson et al. 2024; Igiehon and Babalola 2018; Mendes et al. 2013). Soil textures, for instance, determine microbial assemblages owing to the water retention capacity and respiratory activity of the soil type (Philippot et al. 2013). In carrot systems, a metagenomics study infers that these edaphic variables correlate with differences in microbial composition and distribution, explaining above 70% of the variance (Adebayo et al. 2025). Kim et al. (2017) reported how different soil textures influence carrot susceptibility to certain root‐knot nematodes, *Meloidogyne* spp., suggesting variation in the recruited communities that can enhance the crop resistance. However, this study noted that the sufficient aeration of sandy rhizosphere soil for carrot resilience is limited to bed‐soil and sand mixture trials in the greenhouse, thereby reinforcing the need for further field‐level validation.

### 4.2 Climate Conditions

Climatic variables, such as precipitation, temperature, and humidity, alter rhizosphere conditions, including moisture, thermal regime, and redox potential, which influence microbial community structure and activities (Yang et al. 2024). Because moisture content strongly influences microbial activity and nutrient cycling, aeration and rainfall regimes affect rhizosphere composition (Zou et al. 2024). Similarly, soil temperature alters microbial metabolic rates and capacities due to differing thermal optima across taxa (Philippot et al. 2021). Drought and deficit irrigation commonly reduce microbial biomass and reshape community structure, diminishing nutrient availability (Agunbiade and Babalola 2024; Philippot et al. 2021). Conversely, denitrifiers and anaerobic metabolisms are more favoured under excessive soil moisture, sometimes fostering water‐related pathogens (Zou et al. 2024). While there are relatively scarce climate‐response experiments on carrots, existing studies indicate environmental variable‐driven shifts with only inferred functions (Bagci et al. 2023). These functions are not yet consistently linked to crop performance, highlighting the need for climate‐microbiome studies with carrot systems.

### 4.3 Agricultural Practices

Management and farming strategies that can alter soil components and structure, including cropping systems, fertilisation regimes, tilling, and irrigation, distinctly affect rhizosphere microbiomes in plants (Meena et al. 2023; Rachwal et al. 2021; Schmidt et al. 2019). These strategies influence the community structure and functions by altering the soil properties, impacting the habitat (Wang, Yan, et al. 2022). The effects have been reported both in broad meta‐analyses across different crops and a few emerging carrot‐specific studies, which still require robust field validations (Bagci et al. 2023; Guo et al. 2020; Kracmarova et al. 2022).

#### 4.3.1 Crop Rotation and Cover Cropping Systems

Crop rotation and cover cropping, when implemented, can increase microbial diversity and network complexity in farming systems, which may enhance functional redundancy and disease suppression (Kracmarova et al. 2022; Zhang et al. 2025). Both approaches alter the root inputs and residue quality, causing temporal shifts in rhizosphere community structure or promoting the abundance of particular microbial taxa (Kong et al. 2025). Legume‐based rotation commonly increases N availability, which tends to increase N‐cycling taxa abundance, while cover crop systems can contribute to the carbon inputs that restructure microbial assemblages in the plant rhizosphere (Enagbonma et al. 2025; Seitz et al. 2024). A recent carrot‐specific study shows that legume precrop relatively shapes rhizosphere community structure compared to a continuous cropping system, indicating that carrot rhizosphere microbiomes also respond to cropping system effects (Adebayo et al. 2025). However, while such effects are scarcely reported and mostly theoretical, the empirical outcomes on carrot productivity require more replicated field trials.

#### 4.3.2 Fertilisation Regime

Fertiliser type, quantity, and rate strongly alter soil nutrient profiles and influence microbial biomass, activity, and community composition (Guo et al. 2020; Moretti et al. 2024). Organic amendments, particularly those derived from compost and manure, generally increase soil organic matter and often foster diverse microbial abundance and functions (Bagci et al. 2023; Luo et al. 2023). Long‐term intensive inorganic fertilisation can modify soil nutrients and select for copiotrophic taxa, leading to context‐dependent alterations in diversity metrics (Katherasala et al. 2024; Zhang et al. 2022). Notably, existing carrot‐related studies indicate that organic fertilisation has temporary effects on prokaryotic communities in the rhizosphere, underscoring the need for long‐term trials (Bagci et al. 2023).

#### 4.3.3 Tillage Practices

Tillage alters physical structure, air movement, and organic matter distribution patterns, thereby influencing microbial habitats and community structure (Meena et al. 2023; Srour et al. 2020). The soil structure and organic matter are more stable with conservative tillage systems such as reduced or no‐till practices, fostering a stable and diverse microbiome (Li et al. 2020; Meena et al. 2023). Intensive tillage practices break down soil aggregate structures and reduce microbial population and diversity (Srour et al. 2020). Evidence from meta‐analyses indicates that consistent tillage‐based practices influence nutrient availability and shape microbial composition and diversity (Chen et al. 2020; Li et al. 2025), a pattern that remains understudied for carrot systems.

#### 4.3.4 Irrigation Practices

Irrigation regime directly influences the rhizosphere soil moisture contents and redox heterogeneity, affecting the microbial proliferation and activity levels (Ahmad and Li 2021; Kuerban et al. 2023). Over‐irrigation can create anoxic microsites that favour anaerobic metabolisms, such as denitrifiers and methanogens, and may foster certain pathogens like *Pythium* sp. (Li, Niu, et al. 2024). Despite conserving water, deficit irrigation leads to plant and microbial stress, reducing microbial biomass and activity, which may result in shifts in community composition (Zou et al. 2024). Although carrot‐specific reports are limited, both over‐ and under‐irrigation on rhizosphere microbiome across different crops impact plant‐microbe interactions (Kuerban et al. 2023; Li, Niu, et al. 2024; Zou et al. 2024). Optimal irrigation practices, which maintain soil conditions that enhance desirable microbial activity, are system‐specific (Ahmad and Li 2021), underscoring the need for replicate empirical studies for carrot production.

### 4.4 Crop Genotype

Carrots exist in numerous genotypes consisting of different types, varieties, and cultivars, including the wild and cultivated types; experimental and commercial varieties; and Danvers, Nantes, and Imperator cultivars (Wohlfeiler et al. 2021). The genotype, which reflects genetic diversity relevant to root traits and exudate chemistry, influences community composition and associated functions of the microbiomes inhabiting the rhizosphere. This genotype‐dependent root exudate composition is a major driver of rhizosphere microbial recruitment and activity, which has been demonstrated in several crops and a few carrot genotype studies (Anderson et al. 2024; Hua et al. 2024; Pantigoso et al. 2022; Triviño et al. 2023). A paired metabolome‐microbiome study by Anderson et al. (2024) reported that carrot genotype‐dependent exudates selectively attract taxa with putative plant‐beneficial traits and are linked to differences in residue decomposition. Such selective recruitment supports the idea that genotype‐specific root exudate profiles may inform practices that enhance positive crop‐microbe interactions in the rhizosphere (Anderson et al. 2024). However, whether such selection can translate to reproducible agronomic gains still requires experimental validation. An experimental study by Triviño et al. (2023) reported a certain carrot type (genotype 8503) that increases corn residue decomposition and soil β‐glucosidase activity. The genotype effects correlated with the abundance of specific bacterial families, including Rhodospirilliaceae, Chromatiaceae, and Micromonosporaceae, associated with carbon and nitrogen cycling (Triviño et al. 2023). Additionally, genotype‐driven variation in endophytic assemblages has been recorded from roots of carrots, with the *Alternaria dauci*‐resistant genotype showing distinct fungal endophytes from those of the susceptible type. The resistant genotype harbours taxa previously associated with pathogen antagonism, and how they are linked with the carrot resistance requires further validation (Abdelrazek et al. 2020). Furthermore, evidence shows that crop and soil management practices and genotype interactively shape rhizosphere microbiome and root endophyte dynamics as well as their activities in carrot systems (Abdelrazek et al. 2020; Triviño et al. 2023). Therefore, translating genotype‐microbiome interactions into agronomic benefits for carrot health and productivity still requires mechanistic experimentations across soils and management regimes.

## 5 Potential Biotechnological Utilisation of Carrot Rhizosphere Microbiome

Over the past few decades, formulation of microbial resources, such as biofertilizers, biostimulants, and biocontrol agents, has progressed rapidly, largely driven by research on crop rhizosphere microbiomes, whose functional trait validations and field testing are more established (Backer et al. 2018; Romano et al. 2020). Still, there is no single microbial product or formulation with consistent performance across environments; hence, the continuous search for functionally reliable candidates, especially as agriculture faces pressure from biotic and abiotic variability (Bender et al. 2016; Finkel et al. 2017). Emerging research suggests that the carrot rhizosphere harbours microbial communities with traits relevant to nutrient cycling, growth promotion, and pathogen suppression; however, these functional properties remain under‐characterised compared to staple crops (Pantigoso et al. 2022; Zhu et al. 2024). Such limited knowledge creates gaps in exploring different crops for diversified resource development, even as the interest in microbiome‐based agricultural inputs increases. As a result, there are no known carrot‐specific microbial products that have been commercialised yet, mainly because the agronomic potential of its rhizosphere microbial traits is only being described and remains less validated beyond descriptive studies (Adebayo et al. 2025; Triviño et al. 2023). Nonetheless, initial isolate‐level and consortium studies indicate that carrot‐associated microbes can influence root growth, nutrient availability, crop yield, pest tolerance, and resistance to pathogens (Ahamad et al. 2023; El‐Tarabily et al. 1997; Zhu et al. 2024). These traits point to four potential avenues where such microbes could be biotechnologically explored: these include (i) biofertilizers, which support nutrient mobilisation and promote growth; (ii) biostimulants, which enhance root architecture and plant resilience; (iii) biocontrol agents, which suppress pathogens; and (iv) value‐added bioproducts, including enzymes and consortia with industrial and agronomic importance (Aloo et al. 2022; Romano et al. 2020). The potential of these microbes may remain underexplored until their functional traits are mechanistically validated through systemic multi‐site and multi‐season evaluations (Adedayo and Babalola 2023; Aloo et al. 2022). This underexplored status positions carrot rhizosphere microbiome as a promising but still early‐stage frontier for expanding the diversity and functional range of microbial products. Subsequent subsections summarise each potential application, with relation to current carrot‐specific evidence and limitations, highlighting key research needs for realistic development.

### 5.1 As Biofertilizers

Microbial biofertilizers designed to enhance nutrient availability are among the leading biotechnological applications of rhizosphere research (Romano et al. 2020). Such formulation may contain beneficial strain(s) of bacteria and fungi from the rhizosphere that can improve soil nutrients for plant use and increase crop yields, reducing reliance on chemical/synthetic fertilisers (Aloo et al. 2022). Notably, a recent study on carrot rhizosphere identified *Bacillus firmus* MN3, *Acinetobacter pittii* MP41, and *Bacillus subtilis* PK9 strains with nitrogen‐fixing, phosphate‐solubilising, and potassium‐solubilising potential, respectively. A test consortium of these isolates (N3P41K9) improved carrot growth and increased soil nutrient responses in a controlled, single‐site field experiment (Zhu et al. 2024). Additionally, a study by Ahamad et al. (2023) reported the nutrient‐enhancing potential of a rhizosphere‐associated arbuscular mycorrhizal fungus (AMF), *Funneliformis mosseae*, which increased carrot biomass, root colonisation, and nutrient‐linked growth traits. Such promising single‐site, isolate‐level study findings indicate a notable potential of the carrot rhizosphere microbiome, but still require independent replication across locations and seasons. Inoculant efficacy often requires broader validation, as it typically depends on soil type, native microbiota, climate, and environmental conditions, before proceeding with resource acquisition (Pantigoso et al. 2022). The development stage should involve strain selection, formulation, and shelf‐life testing, dose‐dependent evaluation, and independent multi‐site field studies. While genetic engineering and synthetic biology could, in principle, be considered to enhance microbial traits such as N‐fixation, application of such resources must be guided by ecological risk assessment and regulatory approval (Távora et al. 2022).

### 5.2 As Biostimulants

Microbial biostimulants, formulations of microbes that can enhance nutrient use and stress tolerance, operate by secreting bioactive compounds essential for plant performance and health (Fadiji et al. 2022; Sardrodi et al. 2022). Rhizosphere microbes commonly produce metabolites, such as phytohormones, organic acids, volatile organic compounds, and siderophores, which influence plant physiology and biochemical response to stressors (Adedayo and Babalola 2023). Evidence from a carrot‐specific study indicates that rhizosphere AMF can provide biostimulant effects, where *Funneliformis mosseae* increased carotenoid and chlorophyll contents and induced defence‐related enzyme production against nematode infestation. Although its mechanism of action is unknown, co‐application of the carrot‐associated fungus with organic amendment induced the strongest plant responses (Ahamad et al. 2023). While commercial biostimulants are available for other crops, including some vegetables, evidence of consistent enhancement in carrots is limited and trial‐dependent (Tarigan et al. 2022). A small‐scale product trial reported some improvement in nutrient contents, root vigour, and carrot development, but responses were inconsistent (Gavelienė et al. 2021). Therefore, formulating biostimulants with carrot rhizosphere microbiomes will involve characterising the mode of action of candidates, documenting reproducible effects across replicated field trials, with transparent reporting of trial designs and outcomes.

### 5.3 As Biocontrol Agents

Carrot rhizospheres have yielded microbial strains with potential as biocontrol agents, particularly against soil‐borne pathogens, though from greenhouse and laboratory assays (Ahamad et al. 2023). There is recent carrot‐based evidence of rhizobacterial species, including *Lysinibacillus boronitolerans*, *Shouchella rhizosphaerae*, *Bacillus subtilis*, and *Bacillus proteolyticus*, with antagonistic activity against the causative agent of different crop diseases (Sonam et al. 2024a; 2024b). El‐Tarabily et al. (1997) have long ago reported the antagonistic activity of non‐streptomycetes and streptomycetes actinomycetes from carrot rhizosphere against *Pythium coloratum* Vaartaja, a causal agent of cavity‐spot disease in carrots. These rhizospheric actinomycetes exhibited antifungal properties through the production of non‐volatile metabolites and competition for space and nutrients, which reduced disease symptoms in pre‐infested greenhouse soil and enhanced the growth of carrots (El‐Tarabily et al. 1997). While these findings provide insights into the natural anti‐phytopathogenic potential of carrot rhizosphere microbiomes, targeted field studies are required to substantiate their utility as biocontrol agents. Furthermore, integrative assessment of spectrum activity and non‐target effects, and developing formulations from candidates that retain viability in soils, all across sites and seasons, are essential for translating their potential (Backer et al. 2018; Sardrodi et al. 2022).

### 5.4 Producing Other Value‐Added Products

Soil and rhizosphere microbes are widely recognised sources of metabolites, including enzymes and bioactive compounds for biotechnological uses, such as industrial enzymes, biopolymers, and biodegradation agents (Bennett et al. 2023; Choi et al. 2023; Kaushal et al. 2023). Given the evidence that carrot‐associated microbes can produce secondary metabolites, hormones, organic compounds, and antimicrobials (Ahamad et al. 2023; El‐Tarabily et al. 1997), the carrot rhizosphere theoretically represents a promising reservoir for discovery. However, industrial bioprospecting typically relies on well‐characterised, high‐yielding, genomic‐resolved strains with documented metabolic and fermentation data, resources that remain largely limited for carrot‐associated taxa (Adegboye et al. 2021). Currently, most carrot‐specific research is small‐scale, descriptive, and focused on the plant‐microbiome relationship (Adebayo et al. 2025; Ahamad et al. 2023), therefore, it largely lacks systemic screening pipelines for identifying or developing microbial products. These gaps collectively underscore the carrot rhizosphere as an underexplored and largely untapped microbial resource for biotechnological production. Furthermore, translating these microbes and their products for potential use as therapeutics, probiotics, or food‐based applications requires robust safety assessment, genomic characterisation, and regulatory approval (O'Toole et al. 2017). These requirements are consistent with all new microbial biotechnology discoveries, reinforcing why carrot‐associated strains, whose stability, safety, and metabolic profile remain largely unresolved, have not yet entered application pipelines. These considerations underscore the need for fundamental strain‐level profiling of carrot‐derived microorganisms suitable for future development.

### 5.5 Emerging Tools for Optimising Biotechnological Utilisation of Carrot Rhizosphere Microbiomes

Advances in sequencing techniques, shotgun metagenomics, other meta‐omics, and synthetic biology are enhancing our ability to characterise microbial functions and develop bioproducts. The existing metagenomics and isolate‐level evidence has provided initial functional clues, but translation requires linking the functions to plant outcomes, while ensuring ecological safety (Adebayo et al. 2025; Ahamad et al. 2023; Zhu et al. 2024). Gene‐editing and synthetic biology offer laboratory tools to probe functions and improve microbial potential traits; the environmental release of such engineered microbes is, however, subjected to rigorous risk assessment and regulation pathways (O'Toole et al. 2017; Wei and Li 2023).

## 6 Challenges and Research Gaps Limiting Carrot Rhizomicrobiome Exploration

Although rhizosphere‐based innovations are currently of growing interest to agriculture, the study of carrot‐associated microbiomes is still less developed and remain fragmented compared to that of cereals, legumes, and other major crops (Pantigoso et al. 2022; Romano et al. 2020). Most carrot‐specific studies to date have focused on community profiling with valuable descriptions of the microbial structure but have provided limited insights into the mechanistic functional processes, trait expression, and ecological interactions under field conditions (Adebayo et al. 2023; Zhu et al. 2024). This underdevelopment, not due to the absence of scientific potential, reflects the general research trends in which staple crops receive the majority of microbiome‐based investment (Pantigoso et al. 2022). Consequently, the functional diversity, stability, and applicability of carrot rhizosphere microbiomes remain insufficiently evaluated, limiting the translation of emerging findings into validated microbial tools (Paparella et al. 2024). Bridging these gaps requires not only improved mechanistic works but a clear understanding of carrot‐specific biotic interactions, genotype‐driven microbial assembly, and the essentially understudied root endophyte repertoire, another problem complicated by the traditional underfunding of experimental research on vegetable crops.

### 6.1 Complexity of Carrot Biotic Interactions

Several soilborne pathogens and pests, including fungi such as *Pythium coloratum* and root‐knot nematodes like Meloidogyne spp., affect carrots, reducing their yield and altering their root physiology. These stressors may cause changes in microbial composition of the rhizosphere, both individually or in combination (Ahamad et al. 2023; El‐Tarabily et al. 1997). Yet, there are limited carrot‐specific studies that discuss the multitrophic interactions of pests, pathogens, plants, and microbes in the rhizosphere together. The majority of available literature involves single‐stress or unigenotype designs, which limit their ability to clearly disentangle the multitrophic reality under field conditions (Anderson et al. 2024). Genotype‐driven and management‐specific responses to biotic pressure make the process of assessing the associations and causal effects even more complicated (Abdelrazek et al. 2020; Triviño et al. 2023). These complexities cause the functional importance of microbiome shifts viewed under controlled conditions not fully inferred on carrots (Shakeel et al. 2022). Understanding the mechanistic response of carrot microbiomes to biotic stress and mediation of responses is crucial for optimal microbiome‐informed technology.

### 6.2 Genotypic Impact on Microbial Composition

Genotype variation strongly influences the structural composition and diversity of the carrot rhizosphere microbiome, as well as the rate of organic matter decomposition (Anderson et al. 2024). A certain field‐level experiment indicates that carrot genotype enhances the enrichment of specific bacterial taxa related to residue degradation and nitrogen cycling and alters patterns of enzyme activity in the rhizosphere soil (Triviño et al. 2023). However, these findings are based on a limited number of genotypes and a restricted variety of field conditions, which cannot define a universal core microbiome response (Anderson et al. 2024; Romano et al. 2020). Moreover, larger, multi‐site studies that evaluate whether these responses are consistent or context‐specific are scarce for carrots (Paparella et al. 2024). Such genotype‐specific effects make it more difficult to generalise the genotype‐microbiome patterns across carrot germplasm, suggesting that microbiome‐based applications may require genotype‐specific optimisation (Pantigoso et al. 2022).

### 6.3 Limited Characterisation of Endophytes

Carrot roots harbour diverse fungal endophytes, dominated by Ascomycota, with community structure shaped by various factors, including genotype and crop management strategies (Abdelrazek et al. 2020). Despite their potential roles in stress modulation, pathogen interactions, or nutrient cycling, most endophytic taxa associated with carrots remain uncultured and functionally uncharacterised (Paparella et al. 2024). Consequently, the ecological role of these endophytes and their influence on the rhizosphere community remain speculative, as most proposed functions are based on analogy to other host plants rather than carrot‐specific assays (Abdelrazek et al. 2020; Romano et al. 2020). Similarly, their interactions with the rhizosphere community under variable conditions are poorly resolved, a gap that limits our understanding of the whole‐plant microbial networks below ground essential for carrot‐specific resource acquisition (Backer et al. 2018; Pantigoso et al. 2022).

### 6.4 Research Deprioritisation and Underfunding

Carrots receive less research investment compared to staple crops, and this disparity extends towards their rhizosphere studies (Aloo et al. 2022; Backer et al. 2018). Although this trend indicates historically broader research priorities rather than specific neglect of carrots, it reflects the limited attention and funding that carrot‐specific research initiatives may be receiving (Paparella et al. 2024). As a result, carrot rhizosphere microbiome research remains fewer in number, smaller in scale, and less mechanistic, lacking the long‐term, multi‐site trials and coordinated consortia that have driven progress in cereals and legumes (Anbarasan et al. 2024; Liu et al. 2019). These challenges constrain the understanding of their ecological significance, mainly complicated by gaps, including: (i) limited high‐resolution taxonomic and functional characterisation of carrot rhizosphere communities; (ii) insufficient knowledge of carrot‐specific plant–microbe interactions relevant to targeted microbial amendments; (iii) limited data on how soil type, cropping system, and management practices modulate carrot rhizosphere microbiomes; (iv) early‐stage application of advanced multi‐omics approaches to carrot microbiome research; and (v) minimal long‐term or seasonal studies assessing microbiome stability and successional patterns in carrot systems (Adebayo et al. 2025; Paparella et al. 2024). There is therefore a need for sustained and targeted research investment to translate emerging but growing evidence from the carrot microbiome studies into agronomic innovations.

## 7 Future Direction for Exploring Carrot Rhizo‐Microbiome

Exploration of the carrot rhizosphere is a growing research area with conceptual and empirical implications. However, most carrot‐specific studies are still in the initial stage (single‐site or single‐season trials, isolate screens, or amplicon surveys); thus, future studies should prioritise experiments that step beyond descriptive profiling to mechanistic validation and translational preparedness. The following actionable research priorities (ranked) can enhance robust, reproducible research progress towards carrot‐specific microbiome technology.

- Replicated, multi‐site field trials of candidate strains and consortia.

While single‐location greenhouse or field experiments, such as the N3P41K9 consortium study, provide useful proof‐of‐concept, environmental variability that dictates inoculant performance is less considered in most carrot studies (Zhu et al. 2024). Future work should evaluate the functional efficacy and context‐dependency of the carrot‐associated microbiome based on multi‐site, multi‐season trials with appropriate controls, robust analysis, and statistics with detailed reporting.

ii Targeted genotype‐microbiome studies with mechanistic endpoints.

Given that genotype and management practice interactively influence rhizosphere assembly in the carrot rhizosphere, a more mechanistic approach can elucidate their translational readiness (Triviño et al. 2023). Multiple genotypes should be combined with homogenous management across sites to (i) determine consistent taxa across genotypes, (ii) identify root traits or exudates that drive recruitment, and (iii) establish whether genotype‐specific microbiomes exhibit measurably different impact on carrot performance in the field. Including metabolite profiling and targeted functional assays will take such studies beyond correlational descriptions.

iii Link multi‐omics discovery to hypothesis‐driven validation.

Although shotgun metagenomics, metatranscriptomics, and metabolomics provide candidate taxonomy and functions, translating the omics signals into actionable knowledge still requires isolation, trait assays, and trials under controlled, single‐level, or consortium conditions (Pantigoso et al. 2022). Workflows that directly link carrot rhizosphere‐derived omics data to measurable plant outcomes, such as growth, nutrient use efficiency, and disease suppression (Adebayo et al. 2025), should be prioritised.

iv Standardised isolation, formulation and stability testing.

Furthermore, standardised protocols should be developed for isolating, quality assessment, formulating carriers, testing shelf‐life, and delivering (seed coating, soil amendment) any promising strain or consortium from the carrot rhizosphere. Reports on their viability, dose–response, and compatibility with common agronomic practices should be documented to enable reproducibility and regulatory assessment (Alzate Zuluaga et al. 2024).

v Ecological risk assessment and non‐target effects.

The persistence, horizontal gene transfer capacity, and on‐target effects on soil functioning and natural microbiota organisms of the candidate or consortium, before large‐scale deployments, should be determined. Such evaluations should be integrated into multi‐site trials rather than simplified controlled carrot systems. Transparent biosafety and regulatory pathways must be in place when considering genetic modification (Alzate Zuluaga et al. 2024).

vi Socio‐economic and adoption studies.

The actual adoption of a resource is essentially dependent on farmers' perceptions, cost–benefits, and supply chain (Akinyi et al. 2022). Therefore, parallel socio‐economic studies, including cost implications, farmers and market analyses, should be considered to identify the viability and acceptability of carrot‐specific microbial products. These studies help set realistic development milestones, preventing untimely techno‐optimism of such products.

On this background, to enhance novelty in the carrot‐specific microbial resource acquisition, we recommend a stepwise pipeline for translational process: (A) discovery (omics + targeted isolation), (B) trait validation (lab & greenhouse), (C) formulation and stability testing, (D) multi‐site field validation with ecological risk assessments, and (E) socio‐economic evaluation. This pipeline considers risks associated with lab‐to‐field data translation, with priority on robust evidence accumulation.

## 8 Conclusion

Carrot rhizosphere microbiome research is yielding promising initial insights, including taxonomic profiles, genotype effects, and a few isolate‐consortium studies, but the field is still at an exploratory stage, highlighting its underexploited status. The existing and growing evidence supports hypotheses about potential microbiome‐based biofertilizer, biostimulant, and biocontrol applications rather than established universal solutions. However, the translation is limited by the predominance of simplified studies, limited functional validation, and scarcity of long‐term or multi‐environment trials for the carrot‐specific microbiome. To advance from potential to practice, researchers should prioritise reproducible, hypothesis‐driven research that links omics discoveries to mechanistic validation through robust multi‐site field experiments and ecological risk and socio‐economic analyses. By taking a staged, evidence‐based pipeline approach, discovery‐validation‐formulation‐multi‐site testing‐adoption studies, researchers can be intellectually responsible about which carrot‐associated microbes or agricultural practices are worth developing into safe, effective, and scalable agroecological tools.

## Author Contributions

**Alaba Adewole Adebayo:** conceptualization, Investigation, Writing – original draft, and Writing – review and editing. **Olubukola Oluranti Babalola:** conceptualization, Writing – review and editing.

## Funding

This work was supported by the International Centre for Genetic Engineering and Biotechnology, CRP/ZAF22‐93.

## Ethics Statement

The authors have nothing to report.

## Consent

All authors approved the manuscript for publication.

## Conflicts of Interest

The authors declare no conflicts of interest.

## Data Availability

Data sharing is not applicable to this article as no datasets were generated or analysed in the current study.
